# Schisantherin A alleviates non-alcoholic fatty liver disease by restoring intestinal barrier function

**DOI:** 10.3389/fcimb.2022.855008

**Published:** 2022-09-05

**Authors:** Shenglan Yu, Jiarui Jiang, Qinqin Li, Xuan Liu, Zhengtao Wang, Li Yang, Lili Ding

**Affiliations:** ^1^ Shanghai Key Laboratory of Complex Prescription and Ministry of Education Key Laboratory for Standardization of Chinese Medicines, Institute of Chinese Materia Medica, Shanghai University of Traditional Chinese Medicine, Shanghai, China; ^2^ Phamcological Research Department, Shanghai Research and Development Center for Standardization of Traditional Chinese Medicines, Shanghai, China; ^3^ Institute of Interdisciplinary Integrative Medicine Research, Shanghai University of Traditional Chinese Medicine, Shanghai, China; ^4^ Research and Development Department, Xuzhou Wanwusheng Pharmaceutical Co., Ltd., Xuzhou, China

**Keywords:** Schisantherin A, microbiota, intestinal barrier function, inflammation, NAFLD

## Abstract

**Background:**

Non-alcoholic fatty liver disease (NAFLD) is intricately linked to dysregulation of the gut–liver axis, and correlated with intestinal inflammation and barrier disruption.

**Objectives:**

To investigate the protective effects and possible molecular mechanism of Schisantherin A (Sin A) in a high-fat diet (HFD) induced NAFLD mouse model.

**Methods:**

HFD-fed NAFLD mice were treated with the vehicle and 80 mg/kg Sin A every day for 6 weeks. The gut permeability of the NAFLD mice was assessed by intestinal permeability assays *in vivo* and transepithelial electrical resistance (TEER) measurements *in vitro* were also used to evaluate the function of the gut barrier. TLR4 inhibitor was then used to investigate the impact of Sin A in the LPS- TLR4 signaling pathway. Alternatively, the composition of the microbiome was assessed using 16S rRNA amplification. Finally, the experiment of antibiotic treatment was performed to elucidate the roles of the gut microbiome mediating Sin A induced metabolic benefits in the NAFLD mice.

**Results:**

We found that Sin A potently ameliorated HFD-induced hepatic steatosis and inflammation, alleviated gut inflammation, and restored intestinal barrier function. We also observed that Sin A improved gut permeability and reduced the release of lipopolysaccharide (LPS) into circulation and further found that Sin A can suppress LPS-TLR4 signaling to protect against HFD-induced NAFLD. Sin A treatment altered the composition of the microbiome in NAFLD mice compared to vehicle controls.

**Conclusions:**

Sin A is an effective and safe hepatoprotective agent against HFD-induced NAFLD by partly ameliorating gut inflammation, restoring intestinal barrier function, and regulating intestinal microbiota composition.

## Introduction

Non-alcoholic fatty liver disease (NAFLD) has been a universally acknowledged health concern worldwide, and is likely to increase the risk of various complications, including cardiovascular diseases, malignancy, and multiple liver-associated deaths (Hyysalo et al., 2014; Mantovani et al., 2021). NAFLD is characterized by a spectrum of three clinical presentations, namely, steatosis, non-alcoholic steatohepatitis (NASH), and fibrosis, which accounts for increased complications, the aggravated prognosis of NAFLD, and pronounced hepatocellular carcinoma (Nascimbeni et al., 2013). Of note, mounting evidence suggests that NASH is the primary chronic liver disease and has become the leading cause of liver transplantation over the past 10 years. Lifestyle intervention, currently regarded as the main therapeutic option, cannot effectively treat or slow down the progression of NAFLD (Ratziu et al., 2015). Therefore, it is exceedingly urgent to develop an effective therapy for NAFLD.

Previous works have underlined the important roles of the gut microbiome in host metabolism, which could ameliorate high-caloric diet-induced obesity, delay the progression of NAFLD, and improve metabolic health (Aron-Wisnewsky et al., 2020; Chen and Vitetta, 2020). A high-fat diet (HFD) is a potent physiological stimulus that drives the disturbed composition of intestinal microbiota (Netto Candido et al., 2018), which subsequently contributes to the disarrangement of microbial metabolites. HFD-induced dysbiosis is concomitant with an increased production of lipopolysaccharide (LPS), which is accountable for disrupted epithelial integrity and impaired gut permeability (Cani et al., 2008; Stevens et al., 2018). LPS released into the circulation system can influence the progression of NAFLD to NASH by affecting the intestinal barrier function (Carpino et al., 2020). Additionally, LPS has a profound effect on liver lipid profiles, function, and immunity (Schoeler and Caesar, 2019; Ji et al., 2020). The complicated interplay between the gut microbiota, intestinal barriers, and the immune system indicates the existence of a microbiota–liver signaling axis. However, whether the gut microbiome is an effective target for the treatment of NAFLD remains poorly understood. It is also unclear whether the alteration of LPS signaling causes epithelial integrity changes in the intestinal barrier function, which may correlate with drug development efficacy.

Schisantherin A (Sin A), the major active component of *Schisandra chinensis* Fructus, is of great interest due to its hepatoprotective potential, favorable pharmacokinetic profiles, and no apparent toxicity when studied in rodent models (Sa et al., 2015; Hong et al., 2017). Of note, Sin A could potentially change gut microbiota profiling because of its poor bioactivity. Deciphering the complex interaction between Sin A, the gut microbiome, and microbial metabolites is therefore of utmost importance to understand the critical mechanism of action. In the present study, we demonstrated that the mediation of Sin A on the microbiome is an important contributor to NAFLD improvement in HFD-fed mice and a key factor that reverses metabolic dysfunction caused by HFD (increased liver lipid accumulation, exacerbated inflammation, and compromised gut integrity). Moreover, we performed a mechanistic exploration of the microbiota-derived LPS-TLR4 signals that regulate intestinal barrier function during Sin A treatment, necessary for Sin A-induced metabolic alterations. These results uncovered the signaling pathways critical for our understanding of the microbiota–liver signaling axis and suggested that Sin A represents a promising anti-NAFLD agent.

## Materials and methods

### Chemicals and reagents

Schisantherin A (Sin A) powder was obtained from Nanjing Goren Bio-Technology Co., Ltd. (Nanjing, China, CAS 58546-56-8). The high-fat diet (HFD) was purchased from Research Diets Co., Ltd. (D12492, USA). The toll-like receptor (Tlr4) inhibitor, CLI-095, was provided by Med Chem Express Co., Ltd. Shanghai, China (HY-11109; Shanghai, China). Ampicillin Sodium Crystalline (A9518-25G-9), Neomycin Trisulfate Salt Hydrate Biorea& (N6386-5G), Vancomycin HCL (V2002-250MG) and Metronidazole Bioxtra (M1547-5G) were purchased from Sigma-Aldrich Co., Ltd. (USA). Primary antibody Occludin (ab216327) was purchased from Abcam (Cambridge, UK) and Goat anti-Rat IgG(H+L) Secondary Antibody, Alexa Fluor 488 Conjugate(A-11006), was provided by Thermo Fisher Scientific Inc. (NYSE: TMO, USA). In all cases, cell culture wells and reagents were obtained from Corning Incorporated (Corning, NY, USA) or Gibco (Carlsbad, CA, USA).

### Animals and experimental design

Animal experiments were conducted in accordance with the Guidelines for Animal Experimentation of Shang Hai University of Traditional Chinese Medicine (China), and the protocols were approved by the Animal Ethics Committee of this institution.

To investigate the potential protection of Sin A in HFD-induced NAFLD mice, C57Bl/6J male mice (aged 6 weeks) were randomly divided into three experimental groups (*n*=8–10 mice per group) as follows: chow diet group (Chow); HFD group (HFD); and HFD+Sin A group (Sin A). Treatment was started concomitantly with the introduction of HFD and consisted of daily oral doses of Sin A (80 mg/kg) (Nanjing Goren Bio-Technology Co., Ltd, Nanjing, China) for a total of 6 weeks.

To elucidate the function of LPS-TLR4 signaling in the HFD-fed mice, C57Bl/6J male mice (aged 6 weeks) were randomly divided into four experimental groups (*n*=5–8 mice per group) as follows: HFD group (HFD); HFD+Sin A group (Sin A), HFD + CLI-095 group (CLI-095), and HFD + CLI-095 + Sin A group (CLI-095 + Sin A). A TLR4 inhibitor (CLI-095, 2 mg/kg, *i.p.* injection) was administered every day for 6 weeks. Controls were injected daily with the vehicle (10% DMSO in saline). Parallel experiments using the HFD model and Sin A administration were performed over 6 weeks.

To confirm the mediation of the gut microbiota, C57Bl/6J male mice (aged 6 weeks) were randomly divided into four experimental groups (*n*=5–8 mice per group) as follows: HFD group (HFD), HFD + Sin A group (Sin A), HFD + ABX group (ABX), and HFD + ABX + Sin A group (ABX + Sin A). Antibiotics (Ampicillin Sodium Crystalline, Neomycin Trisulfate Salt Hydrate rea, Vancomycin HCL and Metronidazole Bioxtra were purchased from Sigma-Aldrich, Shanghai, China) were administered in drinking water *ad libitum* and replaced with freshly prepared cocktails every second day. Parallel experiments using the HFD model and Sin A administration were performed over 6 weeks.

### Measurements of serum/hepatic biochemistry

Serum or hepatic levels of alanine aminotransferase (ALT), aspartate aminotransferase (AST), free fatty acids (FFAs), low-density lipoprotein cholesterol (LDL-C), high-density lipoprotein cholesterol (HDL-C), triglyceride (TG), and total cholesterol (TC) were measured using corresponding assay kits (Nanjing Jiancheng Bioengineering Institute, Nanjing, Jiangsu, China) according to the manufacturer’s instruction.

### Tissue histology

For hematoxylin–eosin (H&E) staining, liver and ileum tissues were fixed in a 10% phosphate-buffered formalin, and paraffin was embedded. A tissue section of 5-μm thickness was cut and then stained with H&E according to a standard protocol. Immunofluorescence detection of Occludin was performed on the paraffin section using a standard technique as follows: blocking with 5% BSA at RT for 1 h; overnight incubation with primary rabbit anti-Occludin antibody (ab216327, Abcam, Cambridge, UK) at 4°C; and incubation with secondary antibody (Goat anti-Rat IgG(H+L), Alexa Fluor 488 conjugate, Thermo Fisher Scientific, A-11006, 1:500) at RT for 45 min.

### Oil Red O staining

The hepatic lipid accumulation was evaluated by Oil Red O (ORO) staining assay. Briefly, a liver fraction was washed 3 times with PBS buffer and then was transferred to ice incubation with 10% formalin. After 30 min of fixation, the hepatic tissues were stained with 100 μl of 0.5% Oil Red O suspended in a water solution for 20 min, then transferred to 75% ethanol for 2 min. Lastly, after being rinsed in 100 μl of distilled water for 3 times, the lipid accumulation of tissues was visualized by imaging.

### Metabolic cage

At week 5, mice were individually placed in metabolic cages to determine oxygen consumption and physical activity using CLAMS (Columbus Instruments, Columbus, OH, USA) according to the manufacturer’s instructions. All metabolic studies required a room temperature of 24°C under a 12-h light/dark cycle. Heat production and Respiratory Exchange Ratio were calculated as described previously (Solon-Biet et al., 2015).

### Cold-induced test

At week 6, mice were subjected to a cold room (4–6°C) without access to food, but with normal water. The rectal temperature was measured every 20 min within 2 h after exposure to the cold.

### Metabolic experiments

The glucose tolerance test was performed in diet-induced obese mice after overnight fasting. After a bolus *i.p.* injection of glucose at 2 g/kg, tail vein blood was immediately collected to measure glucose concentration at the indicated time point. In parallel, an insulin tolerance test was performed in 5-h fasted mice, and the glucose levels were measured at the indicated time point after *i.p.* injection of human insulin at 0.75 U/kg.

### Gene expression analysis

Total RNA was extracted using TRIzol reagent (Takara Bio, Inc., Shiga, Japan; #3732) according to the manufacturer’s instructions. RNA concentrations were equalized and converted to cDNA using a kit (Takara; #RR037). Gene expression was measured by qPCR (Applied Biosystems, Thermo Fisher Scientific, Carlsbad, CA, USA) using the SYBR Green system (Takara Bio, Inc.). Expression was normalized to glyceraldehyde-3-phosphate dehydrogenase (GAPDH) or β-Actin. Primer information is provided in **Supplementary Table S1**. All gene expression procedures, including the design of primers, quantitation methods, and validation of PCR environment, were conducted according to previous studies (Johnson et al., 2014).

### Intestinal permeability assay

At week 6, mice were subjected to an administration of Dextran-FITC in the morning. Blood (30 μl) was collected from the tail in 4-h fasted mice. After 15 min of centrifugation, 15 μl of serum from each sample was obtained, then diluted with water to 100 μl. Using a black bottom 96-well plate, the concentrations of Dextran were determined at 525 μm according to the standard curve method.

### Endotoxin measurement

The changes in endotoxin in serum and feces were measured using an LPS enzyme-linked immunosorbent assay (ELISA) kit. In all cases, samples were performed under pyrogen-free conditions and inactivated for 10 min at 70°C. The preparation of fecal samples was performed following a standard protocol (Kim et al., 2012). Finally, endotoxin was measured with a modified kit protocol.

### 16S rRNA sequence

Using fresh and frozen stool samples, gut microbiota was characterized. Total DNA for the gut microbiome analysis was extracted from 200 mg of feces following a standard protocol (Chen et al., 2018). The 16S rRNA gene high-throughput sequencing was performed using a the Illumina HiSeq PE250 platform, according to standard protocols (Magoč and Salzberg, 2011) by Majorbio Bio-Pharm Technology Co. Ltd. Shanghai, China. To reach the 97% similarity of operational taxonomic units (OTUs), using USEARCH, chimeras were filtered and the remaining sequences were clustered (Edgar, 2013). The RDP classifier was required for assigning a representative sequence of each OTU in the Ribosomal Database Project database. The process of 16S rRNA gene sequence data analysis was performed according to linear discriminant analysis effect size (LEfSe), whose differences among biological groups were determined for significance following nonparametric factorial Kruskal-Wallis rank-sum and Wilcoxon rank-sum tests (Wang et al., 2007).

### Primary hepatocyte isolation and culture

The collagenase perfusion method was performed to obtain primary hepatocytes in this study. Briefly, the wide type C57 BL/6J male mice of 6–8 weeks were used to isolate primary hepatocytes. The liver tissues were digested by a perfusion of collagenase type IV solution, then dissected, minced, and transferred to a culture medium through a 70 μm strainer. Finally, the hepatocytes were collected by a centrifugation method with a 500 g speed. The isolated hepatocytes were resuspended and cultured in William’s E medium including 10% FBS and 1% penicillin-streptomycin (PS).

### Cell culture

Human epithelial colorectal adenocarcinoma (Caco-2) cells were obtained from the Cell Bank of Type Culture Collection of the Chinese Academy of Sciences (Shanghai, China). Cells were cultured in DMEM supplemented with 10% FBS and 1% penicillin-streptomycin at 37°C with 5% CO_2_ in a cell incubator. During the experiment, cells were pretreated with Sin A (50 μm) for 2 h and then were stimulated with LPS (2.0 μg/ml) for 22 h.

### Transepithelial electrical resistance measurement

The Caco-2 cells were seeded into Corning Costar™ Transwell™ Permeable (Corning, NY, USA) Supports in 24-well plates at a cell density of 2×10^5^ cells/cm^2^. In all cases, the incubation was 14 days until a monolayer of confluency was reached. Using an EVOM meter, all transepithelial electrical resistance (TEER) measurements were determined at 37°C, following standard protocols (Li et al., 2010).

### Statistical analysis

The results of the biological assay are presented as means ± SD. The differences between the two groups were analyzed by Student’s *t*-test. Results that involved more than two groups were assessed by one-way or two-way analysis of variance (ANOVA) followed by Tukey’s multiple comparison test. All statistical significance was calculated using GraphPad Prism 7 (LaJolla, CA, USA) as follows: a *p*-value of less than 0.05 (*p < 0.05, **p < 0.01, and ***p < 0.001) was considered statistically significant. The statistical methods and corresponding *p* values for the data shown in each panel are included in the figure legends.

## Results

### Sin A exhibited robust attenuation of hepatic steatosis in HFD-fed mice

**Figure 1:**
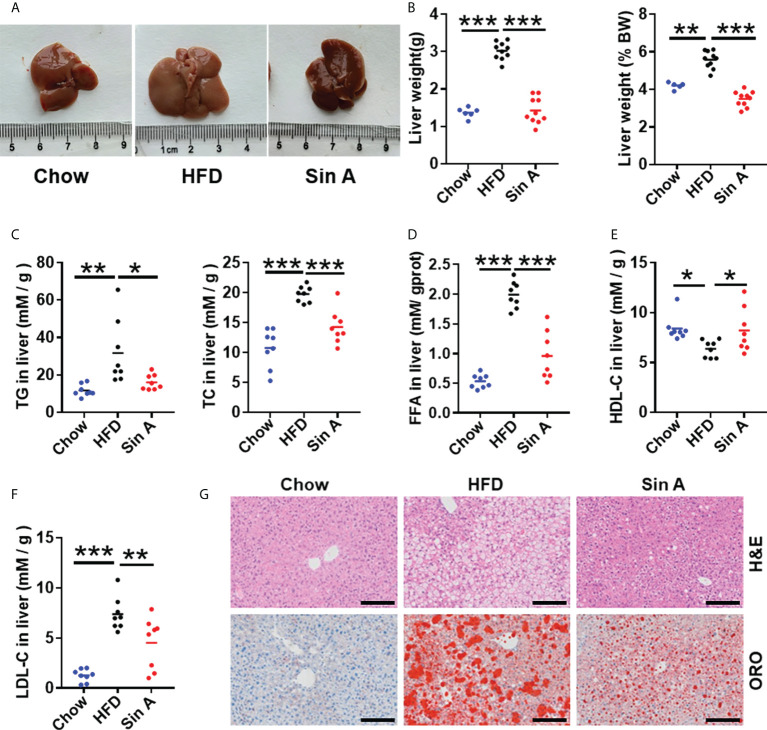
Sin A attenuates hepatic steatosis in HFD-fed mice. Mice were randomly divided into three groups (*n*=8–10). Chow diet-fed mice were orally treated with normal diet (chow). HFD-fed mice were orally treated with vehicle (HFD) or Sin A (80 mg/kg) for 6 weeks. **(A)** Representative images of liver from chow, HFD, and Sin A groups. **(B)** The result of liver weight and the ratio of liver-to-body weight (BW) (*n*=6–10 per group). **(C)** The changes in hepatic triglycerides (TG) and total cholesterol (TC) (*n*=8 per group). **(D)** Free fatty acids (FFAs) contents of liver fraction (*n*=8 per group). **(E,F)** Changes in liver HDL-C, LDL-C level. **(G)** Representative images of H&E staining and Oil Red O staining of liver. Scale bars 100 μm. Values represent mean ± SD. Significant differences were determined by one-way ANOVA for multiple- group comparisons. p*<0.05; **p<0.01; ***p<0.001. TG, triglyceride; TC, total cholesterol; LDL-C, low-density lipoprotein cholesterol; HDL-C, high-density lipoprotein cholesterol; FFA, free fatty acid; ORO, Oil Red O staining.

To address whether Sin A displayed protective effects for a liver metabolic phenotype and inflammation, we used HFD-fed mice as a NAFLD model (Kakimoto and Kowaltowski, 2016) (**Supplementary Figure 1A**). In HFD-fed mice, a 16-week treatment of HFD significantly increased liver size, liver weight, and the ratio of liver weight to body weight. This effect was significantly reversed with a 6-week administration of Sin A (**Figures 1A, B**). According to a previous study, NAFLD is characterized by excessive intrahepatic triglyceride (IHTG) content, which is universally associated with obesity and can lead to inflammation and fibrosis (Lu et al., 2018; Smith et al., 2020; Bril et al., 2020). We found that Sin A significantly decreased the levels of triglyceride (TG), total cholesterol (TC), low-density lipoprotein cholesterol (LDL-C), free fatty acid (FFA), and increased high-density lipoprotein cholesterol (HDL-C) compared with the vehicle controls (**Figures 1C–F**). Consistent with these alterations on lipid profiles, the results of H&E and Oil Red O staining (ORO) also suggested that more lipid droplets occurred in the HFD-fed mice than the control mice, whereas Sin A treatment improved the phenotype of liver in obese mice. (i.e., reduced macrosteatosis, hepatocyte ballooning, and IHTG contents) (**Figure 1G** and **Supplementary Figure 1B**). In summary, these results showed that Sin A performed robust metabolic protection against NAFLD in HFD-fed mice.

### Sin A performed beneficial alteration on hepatic lipid metabolism and inflammation response

**Figure 2:**
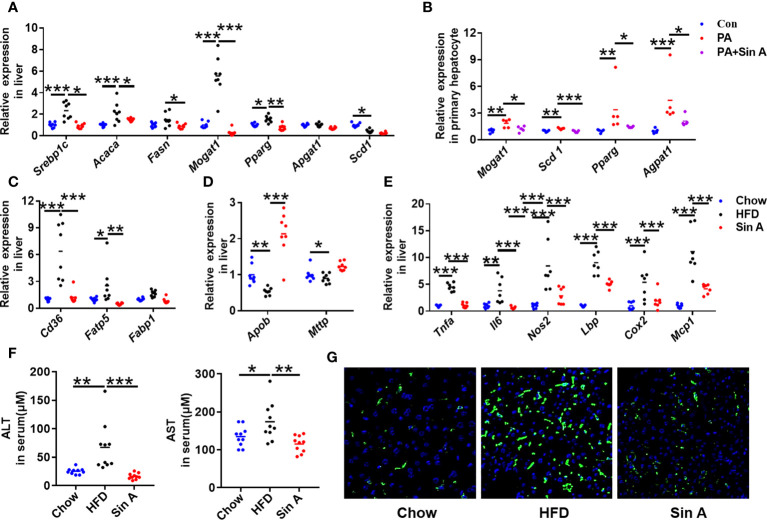
Sin A attenuates hepatic lipid accumulation and inflammatory response in NAFLD mice. Chow-fed mice were treated daily with normal diet (Chow). HFD-fed mice were orally treated with vehicle (HFD) or Sin A (80 mg/kg, Sin A) for 6 weeks. **(A)** The key gene expression associated with fatty acid biogenesis in liver. **(B)** Fatty acid biogenesis-related genes in primary hepatocytes with palmitic acid or Sin A incubation for 24 h (*n*=5 per group). The gene expression associated with fatty acid uptake **(C)** and LDL-C export **(D)** in liver sections. **(E)** The expression levels of inflammation response-related genes in liver (*n*=6 per group). **(F)** Changes in serum chemistry (ALT and AST) of mice (*n*=10 per group). **(G)** The representative images of F4/80 staining in the liver (Scale bars 100 μm). Values are expressed as means ± SD. Statistical significance were determined by one-way ANOVA for the multiple-group comparisons. p*<0.05; **p<0.01; ***p<0.001. Srebp-1c, sterol regulatory element binding protein-1c; Acaca, acetyl-CoA carboxylases alpha; Fasn, fatty acid synthase; Mogat1, Monoacylglycerol O-acyltransferase 1; Pparg, peroxisome proliferative activated receptor, gamma; Scd-1, stearoyl-coenzyme A desaturase-1; Cd36, cluster of differentiation 36; Fatp5, fatty acid transport protein 5; Fabp1, fatty acid-binding protein1; Apob, apolipoprotein B; Mttp, microsomal triglyceride transfer protein; Tnfa, tumor necrosis factor α; Il6, interleukin 6; Nos2, Nitric Oxide Synthase2; Lbp, Lps-binding protein; Cox2, Cyclooxygenase-2; MCP-1, Monocyte chemoattractant protein-1; ALT, Alanine Aminotransferase; AST, Aspartate Aminotransferase.

To further examine the relationship between NAFLD and lipid metabolism, the key genes associated with lipid profiles were evaluated. As shown in **Figure 2A**; the expression levels of genes related to fatty acid synthesis were significantly suppressed after Sin A administration, especially the sterol regulatory element binding protein-1c (*Srebp1c*), acetyl-CoA carboxylases alpha (*Acaca*), monoacylglycerol O-acyltransferase 1 (*Mogat1*), and peroxisome proliferative activated receptor, gamma (*Pparg*). This alteration of lipid synthesis genes was consistent with that in the primary hepatocytes (**Figure 2B**). Furthermore, fatty acid transport-related genes cluster of differentiation 36 (*Cd36*) and fatty acid transport protein 5 (*Fatp5*) were also significantly reduced in Sin A-treated mice when compared with vehicle- treated NAFLD mice (**Figure 2C**). The changes in very low-density lipoprotein export mRNA levels implied that more LDL-C accumulated in the liver of the HFD group compared with the control group and was significantly reversed by Sin A treatment (**Figure 2D**). To understand the close relationship between NAFLD and chronic inflammation (Furman et al., 2019; Farzanegi et al., 2019), the expression levels of specific inflammatory mediators were analyzed by quantitative real-time PCR. The result showed that Sin A was efficacious in reducing the liver inflammatory response as indicated by markedly suppressed expression of tumor necrosis factor α (*Tnfα*), interleukin 6 (*Il6*), nitric oxide synthase2 (*Nos2*), and Lps-binding protein (*Lbp*) (**Figure 2E**). At the same time, Sin A also exerted protective effects against HFD-driven liver damage, as observed by significantly reduced serum levels of alanine aminotransferase (ALT) and aspartate aminotransferase (AST) (**Figure 2F**). Finally, we also evaluated the effects of Sin A on hepatic macrophage infiltration using F4/80 staining. We found that Sin A could remarkably suppress macrophage recruitment in the liver when compared with HFD mice (**Figure 2G** and **Supplementary Figure 1C**). The results above underlined metabolic improvements of Sin A in NAFLD, as validated by greatly ameliorated liver steatosis and reduced inflammatory response.

### Sin A restored intestinal inflammation and gut barrier function in NAFLD mice

**Figure 3:**
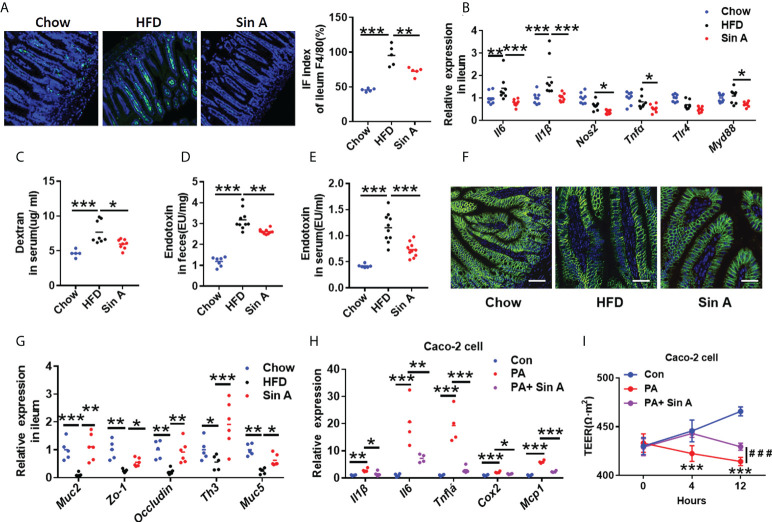
Sin A attenuates gut inflammation and restores intestinal barrier function. Chow-fed mice were treated daily with normal diet (Chow). HFD-fed mice were orally treated with vehicle (HFD) or Sin A (80 mg/kg) for 6 weeks. **(A)** Representative images of F4/80 staining of ileum tissue (Scale bars 10 μm), Right: IF index of ileum F4/80 (%) (*n*=5 per group). **(B)** The changes in inflammation response-associated genes in ileum (*n*=8 per group). **(C)** Changes in dextran concentration in blood 4 h after Dextran-FITC gavage in HFD and Sin A mice (*n*=8 per group). Results of endotoxin in **(D)** feces and **(E)** serum of mice (*n*=10 per group). **(F)** Immunofluorescence for occluding-1 protein in ileum sections. Scale bars 10 μm. **(G)** Results of gene expression related to ileum tight junction molecules (*n*=5–8 per group). **(H)** Results of Qpcr associated with inflammatory mediators in Caco-2 cell after LPS and Sin A incubation for 24 h. **(I)** Changes in transepithelial electrical resistance (TEER) in control, LPS, and LPS+ Sin A groups for the indicated times. Values represent mean ± SD. Significance was determined by Student’s *t*-test for the two-group and one-way ANOVA for the multiple-group comparisons. p*<0.05; **p<0.01; ***p<0.001. Il6, interleukin 6; Il1β, interleukin 1 beta; Tlr4, toll-like receptor4; Myd88, myeloid differentiation 88; Muc2, mucin2; Zo-1, zonula occludens-1; Th3, Muc5, mucin5; Tnfa, tumor necrosis factor α; Cox2, Cyclooxygenase-2; Mcp-1Monocyte chemoattractant protein-1; TEER, transepithelial electrical resistance.

Increased gut inflammation and compromised intestinal barrier function are increasingly emerging as the primary cause of NAFLD progression, which is commonly associated with enhanced translocation of gut microbiota metabolites (Canfora et al., 2019; Ji et al., 2019). As expected, the result of ileum tissue H&E staining showed that vehicle-treated NAFLD mice displayed a pronounced epithelial disruption compared to control mice, which could be modestly restored by Sin A treatment (**Supplementary Figure 1D**). It is well-established that gut macrophage infiltration is the leading cause of intestinal inflammation by secreting inflammatory cytokines and recruiting other inflammation-driven cells. After a 6-week treatment with Sin A, we observed a significant decrease in macrophage infiltration compared with vehicle controls (**Figure 3A**). Consistently, Sin A administration significantly decreased mRNA levels of inflammatory cytokines (*Il6*; *Il1β*, interleukin 1 beta) (**Figure 3B**). We further evaluated the impact of Sin A on intestinal barrier function by utilizing an intestinal permeability assay. Sin A significantly reduced the concentration of Dextran-FITC in the HFD groups (**Figure 3C**). To assess whether Sin A could influence the release of LPS into circulation, which is closely related to epithelial integrity and gut permeability (Stephens and von der Weid, 2020; Longo et al., 2020), we evaluated the levels of fecal and serum endotoxin. As expected, Sin A treatment significantly reduced the production and release of endotoxin in HFD mice compared to vehicle controls (**Figures 3D, E**). Consistently, the restoration of gut barrier integrity in Sin A-treated mice was confirmed by an obviously increased expression of ileum tight junction molecular Occludin (**Figure 3F** and **Supplementary Figure 1E**) and increased mRNA levels of key intestinal barrier mediators and anti-barrier peptides (**Figure 3G**).

To further elucidate the effect of Sin A on the integrity of -cellular barriers, we used human epithelial colorectal adenocarcinoma cells (Caco-2 cell line) to perform *in vitro* experiments. Caco-2 cells were pretreated with Sin A using pre-determined concentrations for 2 h, then stimulated by LPS (2 μg/ml) for 22 h. As expected, the changes in gene expression indicated that Sin A administration (50 μm), co-incubated with LPS treatment, significantly attenuated the inflammatory response and restored intestinal permeability compared to vehicle controls (**Figure 3H**). We also found that the TEER of the LPS group was significantly decreased compared with that in the control group. However, combination with Sin A remarkably reversed this effect (**Figure 3I**). The heatmap generated by Spearman’s correlation analysis described the correlations between different parameters of NAFLD, including endotoxin, blood lipids, hepatic lipids, dextran concentration, inflammation, and intestinal integrity-related genes in ileum tissue (**Supplementary Figure 2A**). This heatmap demonstrated that the intestinal barrier function was negatively correlated with parameters promoting NAFLD and positively correlated with preventing NAFLD.

### Sin A ameliorated hepatic steatosis and inflammation response by LPS-TLR4 signaling

**Figure 4:**
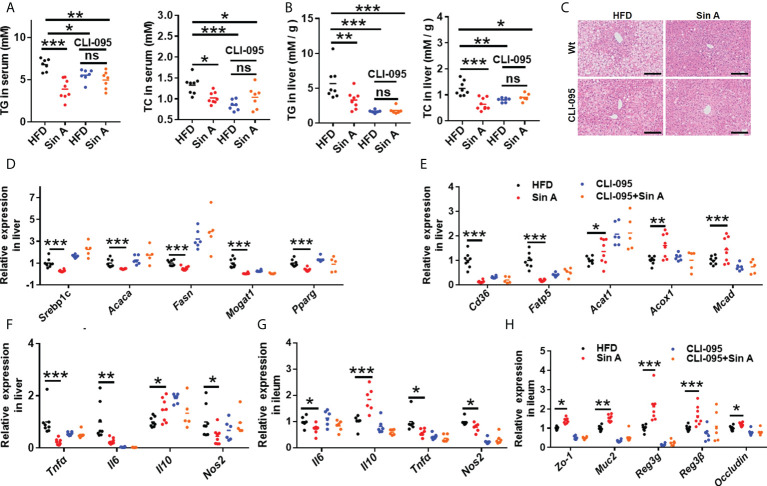
LPS-TLR4 signaling is required for the restoration of Sin A in gut inflammation and intestinal barrier function in NAFLD mice. Mice were randomly divided into four groups (*n*=5–8). HFD-fed mice were orally treated with vehicle (HFD), Sin A (80 mg/kg, Sin A), CLI-095 (2 mg/kg, CLI-095), or CLI-095 plus Sin A (CLI-095+ Sin A) for 6 weeks. **(A)** Changes in TG and TC in serum (*n*=7–8 per group). **(B)** Hepatic TG and TC levels (*n*=7–8 per group). **(C)** Representative images of H&E staining of liver tissue (Scale bars 50 μm). The key gene expression associated with **(D)** fatty acids biogenesis and **(E)** fatty acid uptake and oxidation in liver sections (*n*=5–8 per group). **(F)** Changes in hepatic gene expression related to inflammation response. Results of gene expression associated with **(G)** ileum inflammation response and **(H)** tight junction molecules (*n*=5–8 per group). Values are expressed as means ± SD. Statistical significance was determined by two-way ANOVA for the multiple-group comparisons. p*<0.05; **p<0.01; ***p<0. 001. Srebp-1c, sterol regulatory element binding protein-1c; Acaca, acetyl-CoA carboxylases alpha; Fasn, fatty acid synthase; Mogat1, Monoacylglycerol O-altransferase 1; Pparg, peroxisome proliferative activated receptor, gamma; Scd-1, stearoyl-coenzyme A desaturase-1; Cd36, cluster of differentiation 36; Fatp5, fatty acid transport protein 5; Acat1, cholesterol acyltransferase 1; Acox1, acyl-CoA oxidase 1; Mcad, medium-chain acyl-coA dehydrogenase; Tnfa, tumor necrosis factor α; Il6, interleukin 6; Il10, interleukin10; Nos2, Nitric Oxide Synthase2; Zo-1, zonula occludens-1; Muc2, mucin2; Reg3g, Regenerating islet-derived protein 3 gamma; Reg3β, Regenerating islet-derived protein 3 beta.

As described here, we demonstrated that a circulating increase of LPS impaired the maintenance of intestinal integrity and disrupted metabolic features in NAFLD mice (Carpino et al., 2020). To further investigate the role of LPS-TLR4 signaling in regulating the effect of Sin A on metabolic physiology in NAFLD, we exposed mice to HFD and treated them with the TLR4 inhibitor, CLI-095. HFD mice were evaluated in the following groups: (1) control, (2) Sin A treatment, (3) CLI-095 treatment alone, and (4) CLI-095 + Sin A treatment (**Supplementary Figure 2B**). The CLI-095-treated mice displayed lower lipid accumulation when compared with HFD-fed control mice, while comparable changes were observed in both the CLI-095 and CLI-095 + Sin A treatment groups (**Figures 4A, B**). We also found that Sin A inhibited the LPS-TLR4 signaling pathway to ameliorate hepatic steatosis of NAFLD mice using liver histological staining (**Figure 4C**). To assess whether the loss of TRL4 signaling can influence the lipid profiles, we assessed the changes in specific gene expression in liver sections. Compared to HFD-fed NAFLD mice, Sin A-fed mice displayed a significant decrease in fatty acid synthesis and intake while displaying a significant increase in β-oxidation. These effects could be diminished with the loss of TLR4 signaling (**Figures 4D, E**). qPCR analysis demonstrated that mRNA expression of genes associated with inflammation (*Tnfα*, *Il6*, *Il10*, and *Nos2*) were not changed in mice with inhibited TLR signaling (**Figure 4F**). However, this expression could be significantly suppressed by Sin A in control groups (**Figure 4F**). Strikingly, when the TLR4 signaling was pharmacologically suppressed, Sin A administration neither restored intestinal barrier function nor improved gut inflammation in HFD mice (**Figures 4G, H**). These results suggest that mediation of LPS-TLR4 signaling was required for the beneficial improvements of Sin A on steatosis and inflammation in HFD-fed mice.

### Sin A mediated LPS-TLR4 pathway to reverse metabolic dysfunction in NAFLD

**Figure 5:**
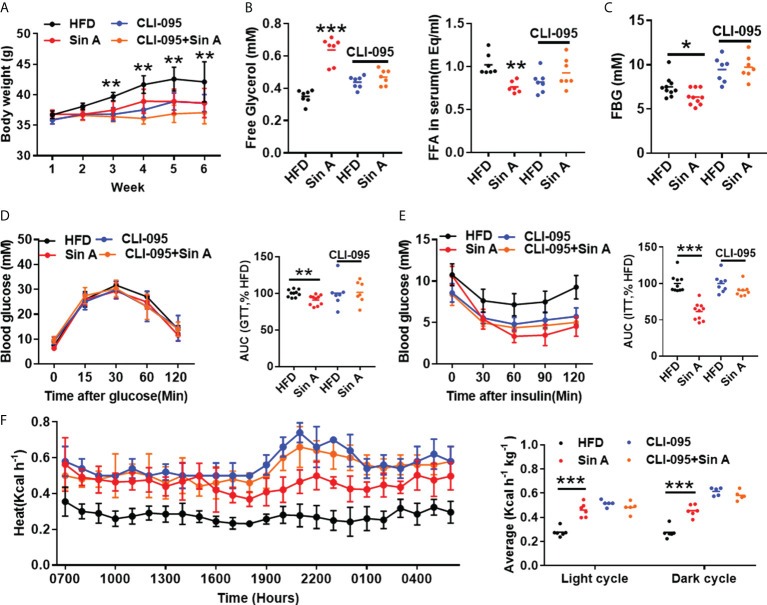
Sin A mediates LPS-TLR4 signaling to ameliorate HFD-induced obesity and insulin resistance in NAFLD mice. Vehicle-group mice were treated with vehicle (HFD) or Sin A (80 mg/kg); CLI-095 group mice were treated with vehicle (HFD) or Sin A (80 mg/kg). **(A)** Body weight evaluation of 6 weeks for HFD, Sin A, CLI-095 and CLI-095 + Sin A groups (*n*=5–8 per group). **(B)** Serum free glycerol levels and free fatty acids (FFAs) (*n*=5–7 per group). **(C)** Fasting blood glucose (FBG) levels (*n*=7–10). Results of **(D)** glucose tolerance test (GTT) and **(E)** insulin tolerance test (ITT) (*n*=7–8 per group). **(F)** Heat expenditure and its respective average of light cycle and dark cycle (*n*=5–6 per group). Values represent mean ± SD. Significance was determined by two-way ANOVA for the multiple-group comparisons. p*<0.05; **p<0.01; ***p<0.001. FFA, fatty fat acid; FBG, fasting blood glucose; GTT, glucose tolerance test; ITT, insulin tolerance test.

Given that NAFLD is accompanied by obesity, and considering that the known impacts of TLR4 signaling in high-fat-induced metabolic disarrangements (Liu et al., 2019; Zhang et al., 2020b), we therefore examined obesity-related parameters in HFD-fed mice in the presence of CLI-095 treatment. Sin A treatment significantly inhibited body weight gain in NAFLD mice, whereas CLI-095 administration abolished this improvement (**Figure 5A**). The effects of Sin A on serum free fatty acids (FFAs) and free glycerol were diminished when TLR4 signaling was blocked in NAFLD mice (**Figure 5B**). In addition, the effect of Sin A on fasting blood glucose (FBG) was remarkably reversed with the loss of TLR4 signaling in mice (**Figure 5C**). At the same time, we observed a potential role for Sin A on LPS-TLR4 signaling with respect to glucose homeostasis, as supported by no significant changes in glucose tolerance and insulin tolerance during CLI-095 treatment (**Figures 5D, E**). The metabolic cage test indicated that Sin A administration exhibited no apparent impact on heat production in CLI-095-treated mice (**Figure 5F**). To confirm this, we also subjected four groups to cold exposure: (1) HFD control, (2) Sin A treatment alone, (3) CLI-095 treatment alone, and (4) CLI-095 + Sin A treatment. We observed that TLR4 signaling is required for the effect of Sin A in the process of energy expenditure, as revealed by no significant changes in fecal temperature observed in both the CLI-095 and CLI-095 + Sin A groups when compared with the control groups (**Supplementary Figure 2C**). Taken together, these data suggested that Sin A mediates LPS-TLR4 signaling pathway to protect against obesity in HFD-induced NAFLD mice.

### Gut microbiota is partly functional in Sin A-mediated metabolic protection in NAFLD mice

**Figure 6:**
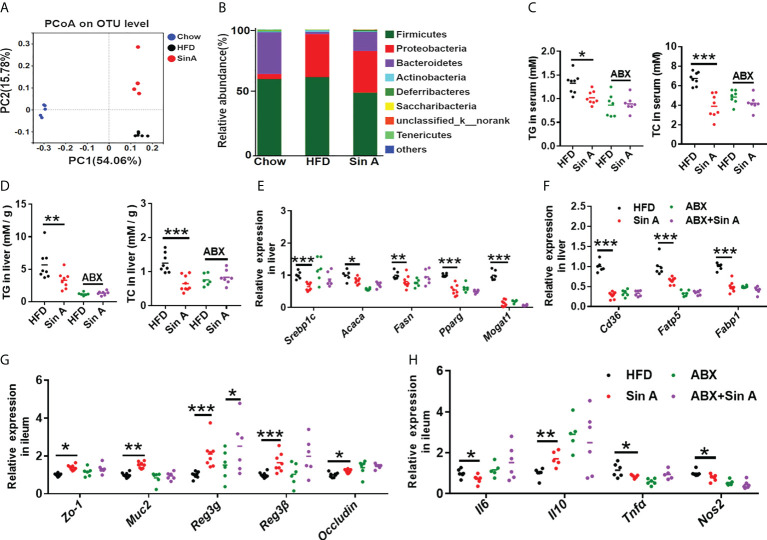
Gut microbiota is functional at Sin A-mediated metabolic protection for NAFLD. Mice were randomly divided into four groups (*n*=5–8). HFD-fed mice were orally treated with vehicle (HFD), Sin A (80 mg/kg, Sin A), antibiotics (ABX), or antibiotics plus Sin A (ABX + Sin A) for 6 weeks. **(A)** Weighted UniFrac PCoA analysis of gut microbiota based on the OTU data of chow, HFD, and Sin A (*n*=5–7 per group). **(B)** Bacterial taxonomic profiling at the phylum level of intestinal bacteria for the above three groups (*n*=5–7 per group). **(C)** Changes in TG and TC in serum (*n*=7–8 per group). **(D)** Hepatic TG and TC levels (*n*=6–8 per group). The key gene expression associated with **(E)** fatty acid biogenesis and **(F)** fatty acid uptake (*n*=5–8 per group). Results of gene expression associated with **(G)** ileum tight junction molecules and **(H)** inflammation response (*n*=5–8 per group). Values are expressed as means ± SD. Statistical significance was determined by two-way ANOVA for the multiple-group comparisons. p*<0.05; **p<0.01; ***p<0.001. PcoA, principle coordinates analysis; ABX, antibiotics; TG, triglyceride; TC, total cholesterol; Srebp-1c, sterol regulatory element binding protein-1c; Acaca, acetyl-CoA carboxylases alpha; Fasn, fatty acid synthase; Mogat1, Monoacylglycerol O-acyltransferase 1; Pparg, peroxisome proliferative activated receptor, gamma; Scd-1, stearoyl-coenzyme A desaturase-1; Cd36, cluster of differentiation 36; Fatp5, fatty acid transport protein 5; Fabp1, fatty acid-binding protein1; Zo-1, zonula occludens-1; Muc2, mucin2; Reg3g, Regenerating islet-derived protein 3 gamma; Reg3β, Regenerating islet-derived protein 3 beta; Il6, interleukin 6; Il10, interleukin10; Tnfa, tumor necrosis factor α; Nos2, Nitric Oxide Synthase2.

It is widely believed that the interplay between host components and the gut microbiome is far from our understanding of their mechanisms (Peng et al., 2019; Huang et al., 2019; Zeng et al., 2020). A great deal of evidence demonstrates that the gut microbiota is the key regulator in the development of NAFLD (Kolodziejczyk et al., 2019; Safari and Gérard, 2019), and targeting the gut microbiome could be a promising therapy for NAFLD. To explore whether Sin A treatment changed the composition of gut microbiota, we performed a 16s rRNA sequence analysis for normal chow-fed, HFD-fed, and Sin A treatment groups. Based on principle coordinates analysis (Pocan) of the weighted Unirac distance, we observed an apparent separation into color-coded clusters among the above three groups (**Figure 6A**), and we also observed an apparent difference in gut microbiota composition after Sin A treatment in the family and genus levels (**Supplementary Figure 3A**). To evaluate the effect of Sin A on the overall composition of the bacterial community, we then evaluated the changes in the degree of bacterial taxonomic similarity at the phylum level. As shown in **Figure 6B**; there were more *Firmicutes* and fewer *Bacteroidetes* observed in the HFD-fed mice as compared with the chow-fed mice, whereas Sin A treatment exhibited obvious protection against these effects. It is possible that HFD-driven gut dysbiosis may cause more production of LPS and then lead to disrupted epithelial integrity, as well as impaired gut permeability, resulting in the increased release of LPS into the circulation system. Therefore, we conducted an antibiotics (ABX) test to investigate whether the mediation of the gut microbiome is responsible for Sin A-induced protection against NAFLD. First, we observed that there was a significant decrease in the LPS level both in the serum and feces of ABX-treated mice compared with vehicle controls (**Supplementary Figures 4A, B**), suggesting that antibiotic treatment may lead to microbiota depletion. ABX-treated mice displayed no significant alterations in lipid accumulation compared with control mice, as revealed by the TG and TC levels in serum and the liver (**Figures 6C, D**). Consistently, there was no apparent effect on the level of gene expression associated with fatty acid synthesis (*Srebp1c*, *Acaca*, *Fasn*, *Pparg*, and *Mogat1*) and uptake (*Cd36*, *Fatp5*, and *Fabp1*) in ABX-treated groups (**Figures 6E, F**). These results demonstrated that ABX treatment can diminish or even abolish the effects of Sin A on lipid metabolism. Second, we explored the roles of gut microbiota in Sin A-induced amelioration of liver inflammation using gene expression performance. We determined that the beneficial effects of Sin A on hepatic inflammation were also dismissed in ABX groups (**Supplementary Figure 4C**). As expected, ABX-treated mice displayed comparable changes in gene expression associated with gut inflammation and intestinal barriers, compared with control mice (**Figures 6G, H**). These data imply that the metabolic benefits of Sin A in NAFLD mice, including improved hepatic lipid accumulation and inflammation along with restored gut barrier function, were partly dependent on the mediation of gut microbiota.

## Discussion

Here, we have demonstrated that 6 weeks of Sin A treatment exhibited remarkable protection against HFD-induced NAFLD in mice. At the same time, we also explored the underlying mechanism of Sin A-mediated metabolic protection against NAFLD, namely, (1) decrease of liver lipid biogenesis and absorption, (2) alleviation of liver inflammation, (3) attenuation of gut inflammation, (3) improvement of gut barrier function, and (4) modulation of gut microbiota dysbiosis.

Non-alcoholic fatty liver disease (NAFLD) has been a leading cause of health concern worldwide, and is highly correlated with cardiovascular diseases, malignancy, and multiple liver-associated deaths (Hyysalo et al., 2014; Mantovani et al., 2021). Hence, it is of great importance to develop an efficient agent to prevent against the progression of NAFLD. In the present study, we demonstrated the efficiency of Sin A in attenuating liver steatosis and inflammation, as well as restoring intestinal barrier function. We found that HFD-fed mice developed NAFLD when compared with chow diet-fed mice, whereas Sin A could apparently improve this condition. Given that lipid accumulation can promote lipotoxicity and trigger hepatocyte death, inflammation, and fibrosis, leading to the progression from NAFLD to NASH (Carpino et al., 2020), we therefore assessed the lipid droplets in the liver, and examined gene expression related to fatty acid synthesis, absorption, and oxidation. Our findings revealed that Sin A treatment significantly inhibited the mRNA levels of hepatic genes, previously functionally linked to lipid synthesis, especially, *Srebp1c*, *Acaca*, *Fasn*, *Mogat1*, and *Pparg*. Considering that inflammation response is highly linked with the progression from NAFLD to NASH, we examined the mRNA levels of hepatic inflammatory mediators, previously functionally linked to inflammation response. As expected, Sin A treatment significantly inhibited the hepatic expression of *Tnfα*, *Il6*, *Nos2*, *Lbp*, *Cox2*, and *Mcp1*, as compared to vehicle controls. Taken together, the efficacy of Sin A on HFD-induced NAFLD mainly involves improvements in hepatic steatosis, inflammatory response, and metabolic disorders.

Gut homeostasis and intestinal barrier function are important regulators in the progression from NAFLD to NASH (Wang et al., 2018; Cui et al., 2019). We observed that both gut and liver inflammation were markedly suppressed in Sin A-treated mice. In particular, the hepatic expression of *Lbp* and *Myd88*, which functions as a direct signal of LPS, was significantly decreased in Sin A-treated mice, suggesting the importance of Sin A in the delay of NAFLD to NASH. Moreover, Sin A treatment dramatically increased the mRNA levels of genes correlated with intestinal epithelial tight junctions, especially *Zo-1*, *Occludin*, and *Mus2*. Of note, the expression levels of regenerating islet-derived protein 3 gamma (*Reg3g*) and regenerating islet-derived protein 3 beta (*Reg3β*), functioning as potent anti-microbial peptides, were increased after Sin A treatment. LPS functions as a potent activator for the TLR4 pathway and the activation of TLR4 signaling is commonly accompanied by impaired gut permeability, which is highly related to the accelerated progression from NAFLD to NASH (Xin et al., 2014; Luther et al., 2015; Song et al., 2017). Thus, growing research has demonstrated that the inhibition of TLR4 signaling plays positive roles in the inflammatory state and epithelial tight junctions (White et al., 2017; Luo et al., 2020; Zhang et al., 2020a). In this study, when TLR4 was knocked down, we did not obserevd apparent improvements on hepatic steatosis, inflammatory response, gut barrier function and obesity in Sin A-treated NAFLD mice. Collectively, Sin A displayed a metabolic protection against NAFLD by mediating the TLR4 signaling pathway.

Gut dysbiosis has been identified as having a key role in contributing to the incidence of NAFLD and the gut microbiome has increasingly emerged as a promising target for NAFLD therapy (Okubo et al., 2018). In this study, we observed an apparent alteration in gut microbial composition between vehicle controls and Sin A-treated mice. We also observed a reduction of *Firmicutes* abundance and an increased level of *Bacteroidetes*, suggesting that Sin A can alleviate HFD-driven gut microbiota dysbiosis. To further determine the mediation of gut microbiota, we performed the ABX experiment to mimic microbiota-depleted status. The results show that ABX-treated mice diminished or even abolished metabolic protection against NAFLD, suggesting the importance of gut microbiota in mediating Sin A-induced metabolic benefits.

Our study had several limitations and potential biases. Although we observed that Sin A can modulate gut microbiome to display protection against NAFLD using the experiment of antibiotics treatment, fecal microbiota transplantation (FMT) experiment is also needed to further confirm the function of the microbiome mediating Sin A-induced metabolic effects in NAFLD mice. Also, we need to further identify which gut microbe functions to affect the LPS-TLR4 signaling pathway, which leads to changes in intestinal inflammation and gut microbiome. We hope that our future work will provide a definitive explanation of the molecular mechanism in immune cells underlying Sin A-induced improvements in NAFLD.

## Data availability statement

The data presented in this study can be found in online repositories. The name of the repository and accession number can be found below: National Center for Biotechnology Information (NCBI) Sequence Read Archive (SRA), https://www.ncbi.nlm.nih.gov/sra, SRR18594304.

## Ethics statement

The animal study was reviewed and approved by Institutional Animal Care and Use Committees (IACUCs) of Shanghai University of Traditional Chinese Medicine, PZSHUTCM200807018. Written informed consent was obtained from the owners for the participation of their animals in this study.

## Author contributions

LD, LY and ZW designed the experiments and secured funding. SY, QL and JJ performed the experiments. SY analyzed the data. SY wrote the manuscript. XL provide the reagent. LD provided editing and final approval of the manuscript. All authors contributed to the article and approved the submitted version.

## Funding

This work is sponsored by the National Natural Science Foundation of China (Grant No. 82122074 and 81773961) to LD and (Grant No. 82130115) to ZW. The present study is supported by the Shanghai Municipal Natural Science Foundation (Grant No. 21ZR1482000) to LD, “Young Qihuang Scholar” project to LY and Graduate Student Innovation Ability Project of Shanghai University of Traditional Chinese Medicine (Y2021060, Y2021050) to SY and QL.

## Conflict of interest

Author XL was employed by Xuzhou Wanwusheng Pharmaceutical Co., Ltd.

The remaining authors declare that the research was conducted in the absence of any commercial or financial relationships that could be construed as a potential conflict of interest.

## Publisher’s note

All claims expressed in this article are solely those of the authors and do not necessarily represent those of their affiliated organizations, or those of the publisher, the editors and the reviewers. Any product that may be evaluated in this article, or claim that may be made by its manufacturer, is not guaranteed or endorsed by the publisher.
