# Supplementary material for: Schisantherin A alleviates non-alcoholic fatty liver disease by restoring intestinal barrier function
Source: Front Cell Infect Microbiol. 2022 Sep 5;12:855008. doi: 10.3389/fcimb.2022.855008 (PMC9483129; doi:10.3389/fcimb.2022.855008)
Supplement: Supplementary file 1 [file DataSheet_1.docx]

**Sin A improves non-alcoholic liver disease partly through**

**restoration of intestinal inflammation and barrier disruption**

**Supplementary figure 1**

1. Study design of Sin A’s impacts on HFD-induced NAFLD mice. (B) NAFLD

activity score based on hepatic steatosis analysis of three groups via Oil red staining assay (n=3-5 per group). (C) IF index of liver F4/80 staining (%) (n=5 per group). (D) Representative images of H&E staining of ileum tissue (Scale bars 50μm). (E) IF index of ileum occluding staining (%) (n=4-5 per group). Value represent mean ± sd. significance was determined by two-way ANOVA for the multiple-group comparisons. *p<0.05; ** p<0.01; *** p<0.001.

**Supplementary figure 2**

(A)Result of correlation matrix (bold values represent correlation coefficient, *, ** and

*** indicate the associations significant. *p<0.05; ** p<0.01; *** p<0.001). (B) Study design of Sin A’s impacts on LPS-TLR4 signaling pathway. (C) Changes of fecal temperature was measured in indicated time points during cold exposure (n=5-6 per group). Values represent mean ± SEM. significance was determined by Student’s t-test for the two-group and one-way ANOVA for the multiple-group comparisons. *p<0.05; ** p<0.01; *** p<0.001.

**Supplementary figure 3**

(A) The heatmap of 50 OTUs in the level of phylum, family and genus from chow diet, HFD model, and Sin A-treated mice (n=5-7 per group).

**Supplementary figure 4**

(A, B) Results of endotoxin in feces and serum of mice (n=7-10 per group). (C)Levels of hepatic gene expression associated with inflammation response was measured (n=5-6 per group). Values represent mean ± sd. significance was determined by two-way ANOVA for the multiple-group comparisons. *p<0.05; ** p<0.01; *** p<0.001. Il6, interleukin 6; Il10, interleukin 10; Cox2, Cyclooxygenase-2; Tnfa, tumor necrosis factor α; Il1β, interleukin 1beta; Myd88, myeloid differentiation 88; Lbp, Lps-binding protein.
