# Supplementary figures and images for: Schisantherin A alleviates non-alcoholic fatty liver disease by restoring intestinal barrier function
Source: Front Cell Infect Microbiol. 2022 Sep 5;12:855008. doi: 10.3389/fcimb.2022.855008 (PMC9483129; doi:10.3389/fcimb.2022.855008)

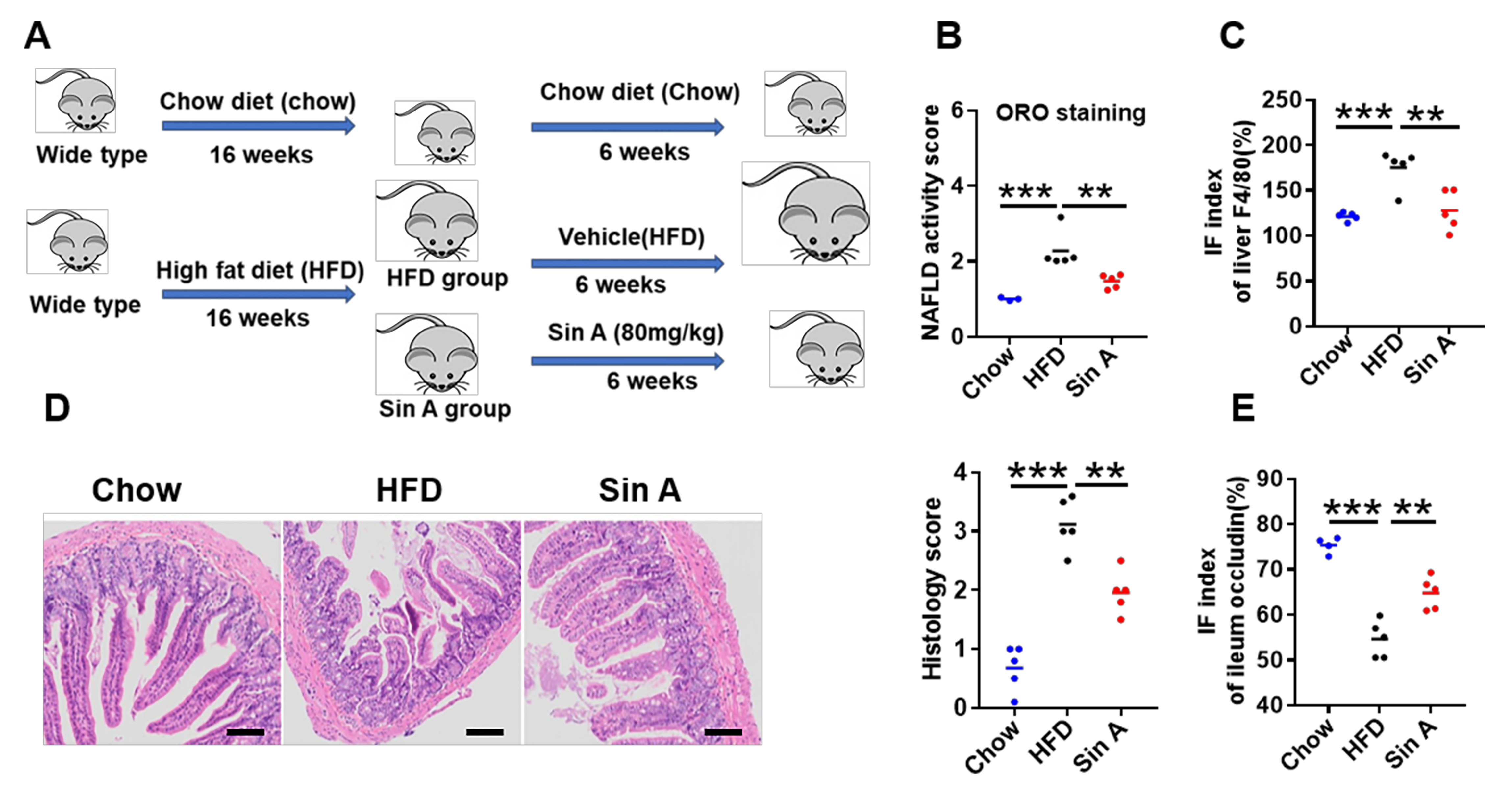

Supplement: Supplementary file 2 [file Image_1.tif]

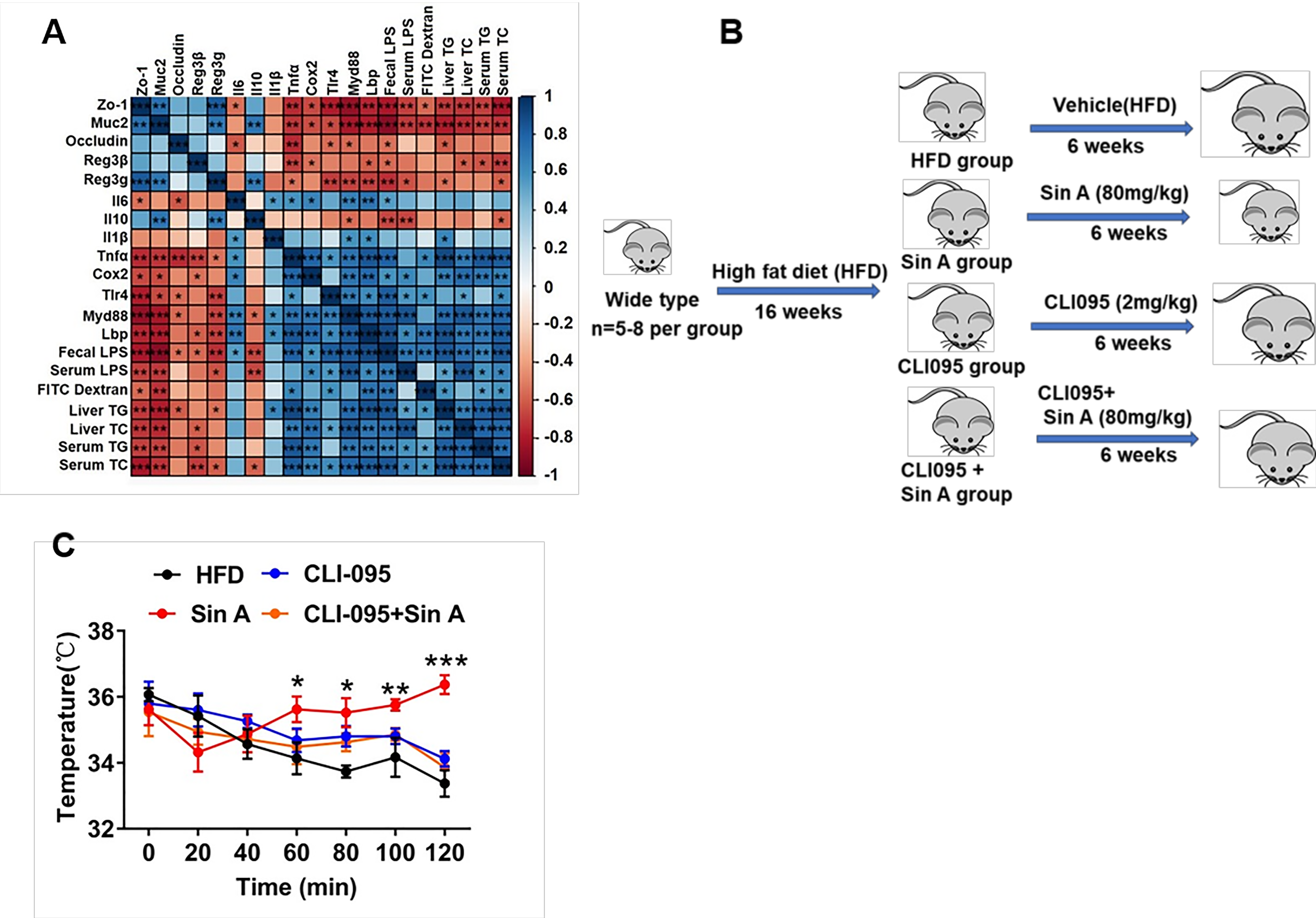

Supplement: Supplementary file 3 [file Image_2.tif]

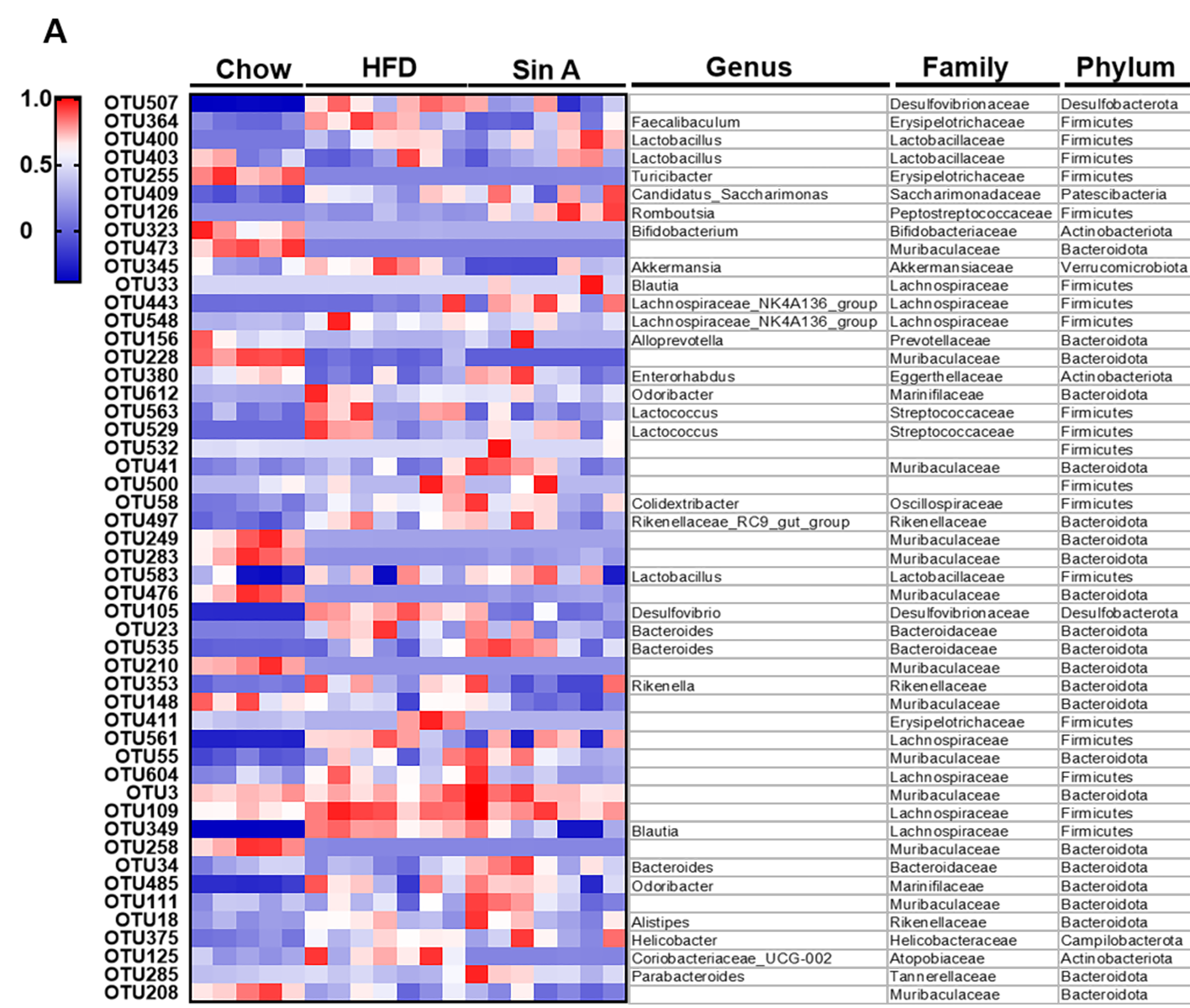

Supplement: Supplementary file 4 [file Image_3.tif]

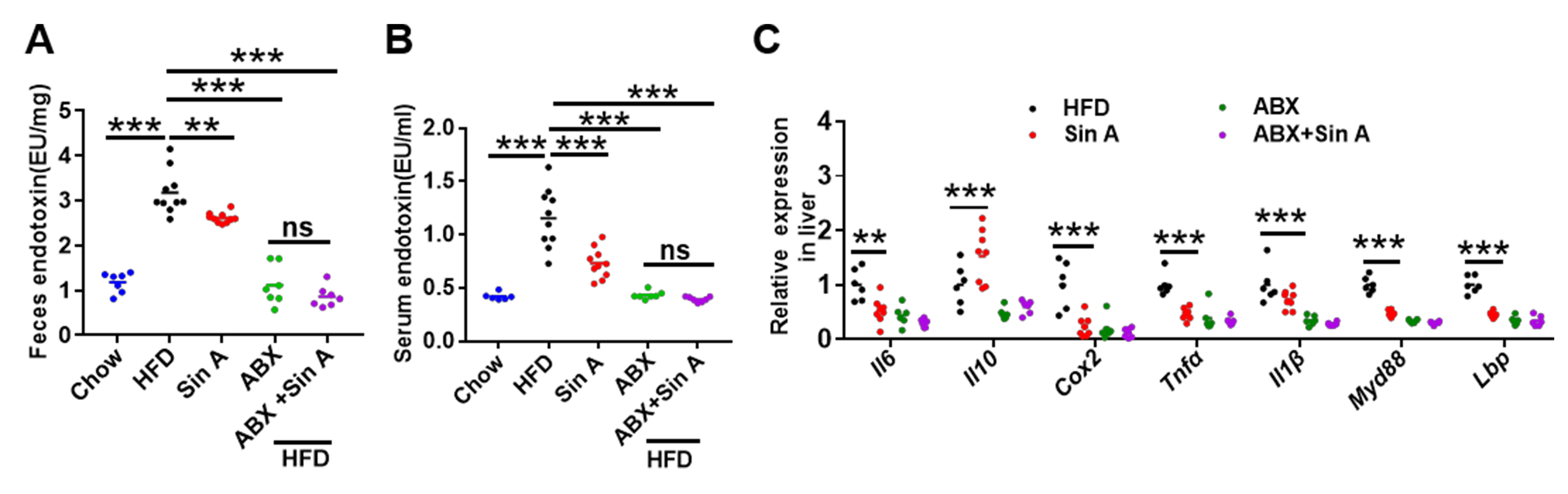

Supplement: Supplementary file 5 [file Image_4.tif]
