# Supplementary material for: Schisantherin A alleviates non-alcoholic fatty liver disease by restoring intestinal barrier function
Source: Front Cell Infect Microbiol. 2022 Sep 5;12:855008. doi: 10.3389/fcimb.2022.855008 (PMC9483129; doi:10.3389/fcimb.2022.855008)
Supplement: Supplementary file 6 [file Table_1.docx]

**Supplementary Table 1.** Primer sequence used for real-time quantitative PCR

| Gene | Primers | Sequence (5’-3’) |
| --- | --- | --- |
| *Srebp1c* | Forward | GGAGCCATGGATTGCACATT |
|  | Reverse | GGCCCGGGAAGTCACTGT |
| *Acaca* | Forward | CAGTAACCTGGTGAAGCTGGA |
|  | Reverse | GCCAGACATGCTGGATCTCAT |
| *Fasn* | Forward | AAGCGGTCTGGAAAGCTGAA |
|  | Reverse | AGGCTGGGTTGATACCTCCA |
| *Mogat1* | Forward | TTGACCCATGGTGCCAGTTT |
|  | Reverse | GTGGCAAGGCTACTCCCATT |
| *Pparg* | Forward | GGTGTGATCTTAACTGCCGGA |
|  | Reverse | GCCCAAACCTGATGGCATTG |
| *Scd-1* | Forward | TAGCCTGTAAAAGATTTCTGCAAACC |
|  | Reverse | GCTGCCGACGGGATCAG |
| *Cpt1α* | Forward | AGCTCGCACATTACAAGGACA |
|  | Reverse | CCAGCACAAAGTTGCAGGAC |
| *Acat1* | Forward | CCCCATTGATTTTCCACTTG |
|  | Reverse | AGCACAACCACACTGAATGC |
| *Acox1* | Forward | CACGGCTATTCTCACAGCAG |
|  | Reverse | CAGGCTGTTAATGTCCACCA |
| *Mcad* | Forward | GGTTTGGCTTTTGGACAATG |
|  | Reverse | TGACGTGTCCAATCTACCACA |
| *Pparα* | Forward | AGAAGTTGCAGGAGGGGATT |
|  | Reverse | TCGGACTCGGTCTTCTTGAT |
| *Fatp1* | Forward | TCTGTTCTGATTCGTGTTCGG |
|  | Reverse | CAGCATATACCACTACTGGCG |
| *Apob* | Forward | TACTTCCACCCACAGTCCCCT |
|  | Reverse | CCTTAGAAGCCTTGGGCACAT |
| *Mttp* | Forward | TCTCACAGTACCCGTTCTT |
|  | Reverse | TCTTCTCCGAGAGACATATCC |
| *Fabp1* | Forward | TGAAGGCAATAGGTCTGCCC |
|  | Reverse | GTCATGGTCTCCAGTTCGCA |
| *Fatp5* | Forward | TTCGAAAGAACCAACCCTTCCT |
|  | Reverse | GCGTCGTACATTCGCAACAA |
| *Cd36* | Forward | GTCAACATATTGGTCAAGCCGC |
|  | Reverse | CCACTCCAATCCCAAGTAAGGC |
| *Tnf-α* | Forward | GCTGAGCTCAAACCCTGGTA |
|  | Reverse | CTCCAAAGTAGACCTGCCCG |
| *Il-1β* | Forward | TTAGTCCTCGGCCAAGACAG |
|  | Reverse | GGCAAGGAGGAAAACACAGG |
| *Il-6* | Forward | GGGACTGATGCTGGTGACAA |
|  | Reverse | ACAGGTCTGTTGGGAGTGGT |
| *Nos2* | Forward | AGCGGCAGCTACTGGGTCAA |
|  | Reverse | ATCCGTCTCGTCCGTGGCAA |
| *Il-10* | Forward | TGCCCCAGGCAGAGAAGCAT |
|  | Reverse | TCAGCCGCATCCTGAGGGTC |
| *Tlr4* | Forward | TGAGGACTGGGTGAGAAATGAGC |
|  | Reverse | CTGCCATGTTTTGAGCAATCTCAT |
| *Myd88* | Forward | TGCCGTCCTGTCTACATCTTTG |
|  | Reverse | GTTGCTCAGGCCAGTCATCA |
| *Lbp* | Forward | CTCTACCCTGATGTCAATGCTG |
|  | Reverse | GAGGTCGTGGAGCTGAATATG |
| *Mus2* | Forward | CCTTGCAGTCAAACTCAAAGT |
|  | Reverse | AAGTTTGCCCCTGGCTATGAC |
| *Muc5* | Forward | GTGGTTTGACACTGACTTCCC |
|  | Reverse | CTCCTCTCGGTGACAGAGTCT |
| *Zo-1* | Forward | GGGAAAACCCGAAACTGATG |
|  | Reverse | GCTGTACTGTGAGGGCAACG |
| *Occludin* | Forward | CCCAGGCTTCTGGATCTATGT |
|  | Reverse | TCCATCTTTCTTCGGGTTTTCA |
| *Reg3b* | Forward | GGCTTCATTCTTGTTGTCCTCCA |
|  | Reverse | TCCACCTCCATTGGGTTCT |
| *Reg3g* | Forward | AAGCTTCCTTCCTGTCCTCC |
|  | Reverse | TCCACCTCTGTTGGGTTCAT |
| *β-actin* | Forward | TGTCCACCTTCCAGCAGATGT |
|  | Reverse | GGGGTCATTGATGGCAACA |
